# Reduction of Elevated Blood Lead Levels in Children in North Carolina and Vermont, 1996–1999

**DOI:** 10.1289/ehp.10548

**Published:** 2008-03-05

**Authors:** Timothy A. Dignam, Jose Lojo, Pamela A. Meyer, Ed Norman, Amy Sayre, W. Dana Flanders

**Affiliations:** 1 Centers for Disease Control and Prevention, National Center for Environmental Health, Division of Emergency and Environmental Health Services, Lead Poisoning Prevention Branch, Atlanta, Georgia, USA; 2 Rollins School of Public Health, Emory University, Atlanta, Georgia, USA; 3 North Carolina Division of Environmental Health, Raleigh, North Carolina, USA; 4 Vermont Childhood Lead Poisoning Prevention Program, Vermont Department of Health, Burlington, Vermont, USA

**Keywords:** blood lead level, capillary, chelation, children, surveillance, venous

## Abstract

**Background:**

Few studies have examined factors related to the time required for children’s blood lead levels (BLLs) ≥ 10 μg/dL to decline to < 10 μg/dL.

**Objectives:**

We used routinely collected surveillance data to determine the length of time and risk factors associated with reducing elevated BLLs in children below the level of concern of 10 μg/dL.

**Methods:**

From the North Carolina and Vermont state surveillance databases, we identified a retrospective cohort of 996 children < 6 years of age whose first two blood lead tests produced levels ≥ 10 μg/dL during 1996–1999. Data were stratified into five categories of qualifying BLLs and analyzed using Cox regression. Survival curves were used to describe the time until BLLs declined below the level of concern. We compared three different analytic methods to account for children lost to follow-up.

**Results:**

On average, it required slightly more than 1 year (382 days) for a child’s BLL to decline to < 10 μg/dL, with the highest BLLs taking even longer. The BLLs of black children [hazard ratio (HR) = 0.84; 95% confidence interval (CI), 0.71–0.99], males (HR_male_ = 0.83; 95% CI, 0.71–0.98), and children from rural areas (HR_rural_ = 0.83; 95% CI, 0.70–0.97) took longer to fall below 10 μg/dL than those of other children, after controlling for qualifying BLL and other covariates. Sensitivity analysis demonstrated that including censored children estimated a longer time for BLL reduction than when using linear interpolation or when excluding censored children.

**Conclusion:**

Children with high confirmatory BLLs, black children, males, and children from rural areas may need additional attention during case management to expedite their BLL reduction time to < 10 μg/dL. Analytic methods that do not account for loss to follow-up may underestimate the time it takes for BLLs to fall below the recommended target level.

Studies reporting an inverse relationship between children’s blood lead levels (BLLs) of 10–24 μg/dL and cognitive function/performance (Bellinger et al. 1991; Ruff et al. 1993) motivated the Centers for Disease Control and Prevention (CDC) to lower the level of concern from a BLL ≥ 25 μg/dL to ≥ 10 μg/dL in 1991 (CDC 1991). Although prevention efforts have resulted in an 86% decline in BLLs ≥ 10 μg/dL in children in the United States from the 1970s to the present, an estimated 310,000 children < 6 years of age still have BLLs ≥ 10 μg/dL (Schwemberger et al. 2005). A key prevention strategy has been to identify children with BLLs ≥ 10 μg/dL and to enroll them in case management to reduce their lead burdens.

Collecting blood lead is considered to be the most useful tool for screening and diagnostic testing (Agency for Toxic Substances and Disease Registry 2005). Blood lead levels correlate most closely with recent environmental exposure. The excretory half-life of lead in adult blood is approximately 36 days (Todd et al. 1996), but estimates in children are longer (Manton et al. 2000). Prolonged exposure, which can come from both internal and external sources, prevents BLLs from declining to lower levels. The most common external source of exposure in children is deteriorated lead-based paint dust (CDC 1997). The most common internal exposure source is from lead reservoirs in bone, which are the largest component of body burden, accounting for 70% of all lead in children, and from where replenishment of lead in the blood occurs. Half-life of lead in bone is usually measured in years or even decades (Graziano 1994).

Although identification of the factors influencing the time to reduce a child’s BLL is essential to improving case management, few studies have investigated this issue. New York City investigators estimate that, on average, it takes 6–12 months for children’s BLLs to decline from ≥ 20 μg/dL to < 10 μg/dL, when medical and environmental management is consistent with CDC recommendations for managing children with elevated BLLs (Matte T, personal communication).

An analysis of Wisconsin surveillance data from 1995 to 1997 showed that BLLs decreased 2.6 μg/dL per year, and the mean time for decline among children with confirmed BLLs ≥ 20 μg/dL to drop below 10 μg/dL was just over 4 years (Wisconsin Childhood Lead Poisoning Prevention Program, unpublished data). A study published in 2001 found that those with higher peak BLLs take longer to decline to < 10 μg/dL (Roberts et al. 2001). Specifically, children with BLLs of 25–29 μg/dL required on average 24 months to fall below 10 μg/dL compared with children with BLLs of 10–14 μg/dL, who required 9.2 months. However, those findings may have been biased because the analysis did not take into account the contribution of children whose BLLs did not fall below 10 μg/dL. A more recent study reported times of decline of 11.6 months in the 10–14 μg/dL category and 12.7 months in the 15–19 μg/dL category among 2,109 children ≤ 3 years of age from six states enrolled in case management; however, the authors also excluded children who were censored in their analysis (Whitehead and Leiker 2007).

To assess factors related to the time required for children’s BLLs ≥ 10 μg/dL to decline, we analyzed routinely collected blood lead surveillance data from lead poisoning prevention programs in Vermont and North Carolina. We compared three different analytic approaches: *a*) a central analysis that included censoring children whose BLLs did not fall below 10 μg/dL at the time of their last test; *b*) an analysis that simply excluded children who were censored; and *c*) a sensitivity analysis that inferred the time for children’s BLLs to fall below 10 μg/dL on the basis of linear interpolations between the times of the last measurement above and the first measurement below the level of concern.

## Methods

### Study design and population

The Childhood Lead Poisoning Prevention Branch of the CDC maintains the Childhood Blood Lead Surveillance database, consisting of childhood blood lead surveillance data from 43 states. States considered for inclusion in this study were those that submitted 1996–1999 surveillance data to the CDC. During this period, two of these states (Vermont and North Carolina) submitted data that included at least two tests on the majority (> 90%) of children with BLLs ≥ 10 μg/dL (*n* = 1,368 children). We excluded 292 children who were > 6 years of age during their first elevated screening test, 74 children who had only two total tests, and 6 children whose records indicated that they had received chelation therapy to lower their BLL. We limited our analysis to children < 6 years of age with at least three tests: *a*) an initial elevated capillary, venous, or unknown sample screening test; *b*) an elevated confirmatory venous test (qualifying BLL); and *c*) a follow-up test (*n* = 996). Time to decline began at the qualifying blood lead test. The restriction to use venous samples at the confirmatory test avoids a potential positive bias associated with BLLs resulting from capillary tests (Anderson et al. 2007; Schlenker et al. 1997).

### Data analysis

Kaplan–Meier survival curves were constructed to assess factors associated with shorter time for BLLs to fall below 10 μg/dL. The independent variables of interest were age at qualifying BLL, sex, race, state of residence, and urban/rural status. All factors were coded using indicator variables, except age at qualifying BLL, which was treated as continuous. The qualifying BLL result was categorized into quintiles, so we could compare our results with those of Roberts et al. (2001). We considered children to be from an urban area if they lived in a metropolitan statistical area in Vermont or North Carolina (U.S. Census Bureau 2002); we classified children not living in an urban area as rural. Vermont had three counties and North Carolina had 41 counties categorized as urban. Children who were not classified as white, black, Hispanic, or “missing race” had their race categorized as “other.” We included children not documented to have attained a BLL < 10 μg/dL in the analysis, and censored at the time of their last test. Follow-up stopped at the time of a child’s last test (Kalbfleisch and Prentice 1980). We called this the central analysis.

We used log/log survival curves and extended Cox models to evaluate whether each variable met the proportional hazards assumption. All variables met this assumption except for qualifying BLL. Therefore, we stratified the analysis by qualifying BLL category in summary models and also analyzed associations separately within categories defined by qualifying BLL category. We screened for collinearity by examining correlations between independent variables. No correlation coefficients exceeded 0.5, even within strata of the qualifying BLL categories (results not shown). Backward selection regression was used to identify the most predictive models, with a critical *p*-value of 0.05 used to determine entry into the model. We used likelihood ratio tests to assess potential interactions. Significance was determined at the 0.05 level. There was evidence of statistical interaction between the child’s area of residence and qualifying BLL in relation to the time required for children’s BLLs to decline to < 10 μg/dL (*p* = 0.0374).

We also evaluated the effects that different methods of analyses could have on resulting time to BLLs declining to < 10 μg/dL. We accomplished this by conducting two additional analyses: *a*) to make a comparison with the Roberts et al. (2001) study, we conducted an analysis in which the censored children were excluded; and *b*) we performed an analysis in which the amount of time for a BLL to reach below 10 μg/dL from the time between the last BLL > 10 μg/dL and the first BLL < 10 μg/dL. All analyses were done using SAS version 9.1 (SAS Institute Inc., Cary, NC).

## Results

Among the 996 children in the study, the mean (± SD) initial BLL was 19.6 ± 8.2 μg/dL. The average number of days between the initial and qualifying blood lead tests was 49.6 days. About 77% of the children were from North Carolina, 40% were white, 40% were black, and more than two-thirds of the children were 12–35 months of age—the time when children are at most risk for elevated BLLs (Table 1). The mean age of the children at qualifying BLL was 2.1 ± 1.3 years. Children who were excluded from the study cohort were similar in demographics and initial BLL (data not shown). Males tended to have a higher mean initial BLL compared with females (19.9 μg/dL and 18.8 μg/dL, respectively), but this difference was not significant (*p* = 0.305). Overall, the median time to attain a BLL < 10 μg/dL was 382 days [95% confidence interval (CI), 356–418 days], slightly more than 1 year (range, 22–1,285 days). In the cohort, censored children (*n* = 408) contributed 5 to 1,086 days and uncensored children (*n* = 588) were followed from 22 to 1,280 days. Children who were censored had similar demographics, and on average, had slightly higher initial BLLs than did children whose BLLs dropped below 10 μg/dL.

**Figure 1:**
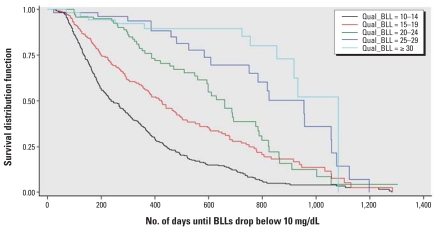
Kaplan–Meier curves of the time required for blood lead levels to drop below 10 μg/dL among children < 6 years of age tested for blood lead from Vermont and North Carolina in 1996–1999, stratified by qualifying (Qual) BLL (μg/dL) (*n* = 996).

We found clear differences in the time required for BLLs to drop below 10 μg/dL based on the child’s qualifying BLL category (Figure 1). As expected, children with higher qualifying BLLs took the longest to reach a BLL < 10 μg/dL when compared with the lower BLL categories. A log-rank test for trend was statistically significant (χ^2^ = −12.2, df = 4, *p* < 0.0001), reinforcing the visual impression of the trend toward longer times of decline among children with higher BLL categories. Children in the BLL category 25–29 μg/dL took > 2.5 years to decline to < 10 μg/dL (median no. of days = 954; 95% CI, 750–1,057) and children in BLL category ≥ 30 μg/dL took 3 years to reach a BLL < 10 μg/dL (median no. of days = 1,083; 95% CI, 917–1,083) (Table 2).

Race, sex, and area of residence were also predictors of the time required for a child’s BLL to drop below 10 μg/dL (Table 3). The median time for BLLs to decline to < 10 μg/dL was approximately 363 days for white children compared with 420 days for black children. Male children had a median time of decline of 400 days compared with 363 days among female children. Children living in rural areas had a median time of decline of 400 days, whereas children living in urban areas required 360 days.

The median time required for black children’s BLLs to decline to < 10 μg/dL took longer than those of all children in the central analysis in each BLL category except in the 20–24 μg/dL BLL category (where it was the same) (Table 2). BLLs of black children consistently required > 33 months to fully decline, regardless of the qualifying BLL category.

When stratified by qualifying BLL category, the analysis in which we excluded censored children yielded the shortest median times for a child’s BLL to decline below 10 μg/dL. The linear interpolation sensitivity analysis yielded median times of decline that were consistently lower in each qualifying BLL category when compared with the central analysis and consistently higher when compared with the analysis that excluded censored children (except in the 10- to 14-μg/dL BLL category) (Table 2).

## Discussion

The BLLs of black children took longer to decline than those of others, regardless of qualifying BLL range. This finding was significant after adjusting for qualifying BLL category and other independent variables. It is not clear from these surveillance data why this racial disparity exists. One possible explanation is that black children are not receiving timely and appropriate follow-up care. Findings from Kemper et al. (2005) provide evidence of differences in follow-up. These researchers found that among Medicaid-enrolled children < 6 years of age with elevated screening tests, follow-up was lower for Hispanic or black children than for white children. A key intervention to reduce children’s BLLs is to identify and remove all sources of lead exposure (CDC 2002). The slower decline in BLLs among black children may reflect a greater length of time to identify and/or remove lead sources.

Another possible explanation for the longer time for black children’s BLLs to decline is that the children may have been exposed to lead for a longer period of time than other children. Previous studies have documented a higher prevalence of BLLs ≥ 10 μg/dL among children who are black (Dignam et al. 2004; Lanphear et al. 1996; Meyer et al. 2003; Pirkle et al. 1998; Raymond et al. 2007; Schwemberger et al. 2005). When ongoing exposures of lead decline, BLLs decline, but lead is also stored in bone (Hu and Hernandez-Avila 2002). Past maternal lead exposures that are stored in bone can be mobilized during pregnancy and lactation and expose the developing fetus. If children are exposed both prenatally and postnatally, this accumulated exposure may take longer to reduce BLLs.

The BLLs of male children took longer to decline than those of female children, after adjustment. It has been established that adult males have higher BLLs than adult females (Counter et al. 2001; Pirkle et al. 1998). This is thought to be attributable to generally higher exposures among men, because men have higher blood hematocrit levels, as lead in blood binds to red blood cells (Vahter et al. 2007), and attributable to the increased skeletal mass of men compared with women (White and Sabbioni 1998). However, in young children differences in mean BLLs between young boys and girls have not been found (Baghurst et al. 1992; Strömberg et al. 2003).

Children from rural counties took longer for their BLLs to decline than children living in urban counties. It is not clear from these surveillance data why this geographic difference exists. Children from rural areas may not always receive timely medical follow-up, home visits, and lead source investigations, possibly because of transportation and other health care access issues. We examined the number of days between the qualifying and follow-up blood lead tests by urban/rural status as a measure of follow-up timeliness. Rural children required a slightly longer time (148.8 days) for follow-up than did urban children (140.9 days), but the difference was not significant (*p* = 0.345). Additionally, children from rural counties tend to live in parts of North Carolina and Vermont with a larger number of older housing as a percentage of all housing, giving families who have children with elevated BLLs fewer choices to relocate to lead-safe housing. For both states combined, the percent of pre-1950 housing in rural areas is 21.6% compared with 13.6% in urban areas.

As expected, we found that the length of time for children’s BLLs to drop below 10 μg/dL depended on the qualifying BLL. Roberts et al. (2001) demonstrated that the time of clearance of toxics from the body without pharmacologic intervention is directly proportional to the peak concentrations of those toxics in the blood. We used the qualifying BLL in our study to categorize the baseline value because these levels were predominantly the peak concentrations observed and were all venous sample types. The efficacy of chelation therapy to reduce the adverse health effects of elevated BLLs has not been demonstrated for BLLs < 45 μg/dL (Liu et al. 2002; Rogan et al. 2001). Although we excluded from our analysis records that indicated that chelation was administered, it is possible that some of the remaining 1.0% of children with BLLs ≥ 45 had been chelated. This may have slightly decreased the overall amount of time observed in this study for children’s BLLs to drop below 10 μg/dL among children with a high qualifying BLL.

The comparison of three different analytic approaches allowed us to assess the impact that censoring had on the estimate of the time to attain the desired BLL. Typically in survival analyses, individuals who do not reach an event (in this case, a BLL < 10 μg/dL) are censored at the end of their follow-up period. Therefore, in our central analysis, the contribution of time from children whose BLLs remained elevated was censored when their last test was recorded. We hypothesized that excluding children whose BLLs failed to return to < 10 μg/dL would lead to an underestimation of the time until BLLs dropped below 10 μg/dL. Even though it was inappropriate to exclude censored children in the analysis, we wanted to compare our results with those of Roberts et al. (2001). When censored children were excluded, we found dramatically shorter times of decline, validating this expectation.

We also observed shorter times of decline compared with the Roberts et al. (2001) study in each BLL stratum except at the 25–29 μg/dL level (and the ≥ 30 μg/dL level, because their study did not have the category), regardless of how we analytically treated children whose BLL did not drop below 10 μg/dL. It is unclear why the study by Roberts et al. (2001), which excluded censored children, reported longer median times of decline. One reason may be the racial composition of the study populations. The Roberts et al. (2001) study comprised 98% African-American children, whereas our study population was equally white and black. A more recent study by Whitehead and Leiker (2007) also reported longer times of decline in the 10–14 and 15–19 μg/dL categories (348 and 381 days, respectively) among children ≤ 3 years of age who were enrolled in case management. However, the authors used analysis of variance and excluded children who did not attain a BLL < 10 μg/dL in their analysis.

In our central analysis we did not know precisely when BLLs declined to < 10 μg/dL, so in additional analyses, we used linear interpolation (sensitivity analysis) to estimate time to this event. These results are more likely to approximate the true time until the desired end point than the central analysis because linear interpolation estimates the time at which children’s BLLs initially decline to < 10 μg/dL.

The large number of children censored before their BLLs fell below 10 μg/dL (40.9%) suggests that many children may be lost to follow-up. Children with elevated BLLs are frequently from lower socioeconomic backgrounds, and follow-up testing may not always occur because of transportation and other health care access issues. In addition, some health care providers and lead poisoning prevention programs may stop providing follow-up services when BLLs fall below 15 μg/dL. In our study we compensated for missing blood lead test results < 10 μg/dL by censoring. A bias could have resulted if the time to decline of BLLs to < 10 μg/dL of those censored differed from that of those children whose BLLs declined to < 10 μg/dL. Similarly, because time until documentation of BLLs to decline to < 10 μg/dL was measured in the central analysis, those results are probably conservative estimates of the true time in this cohort of children.

Another limitation of this study was incomplete data resulting in an inability to control for other important demographic/risk factors that might influence time until BLLs decline to < 10 μg/dL. For example, information on the age of houses, to assess risk for lead paint exposure, was known for only a few children, so its impact could not be fully assessed. Similarly, information was infrequently recorded for Medicaid status (a risk factor for elevated BLLs) and, in a small number of cases, whether chelation therapy was administered. We would have liked to consider these issues in our analysis. We also would have liked to assess whether children with several address changes during the period of analysis were more likely to have longer times until decline or learn of the results of environmental investigations, but detailed address and environmental investigation data were not available. Finally, we did not estimate the biologic half life of lead. We refrained from this approach because we felt that the decline in BLLs reflected not only biologic clearance, but also potentially ongoing or recurring exposures, case management, and other behavioral factors. These are areas that need further investigation.

In conclusion, having a high confirmatory BLL, being black, being male, and living at a rural address are associated with a longer time until BLLs decline to < 10 μg/dL. Further study is needed to understand these and other factors associated with longer times until decline of BLLs to < 10 μg/dL. The CDC recommends more efforts to remove lead-based paint hazards in and around housing before children have elevated BLLs (CDC 2002). Targeted efforts to prevent further lead exposure and aggressive case management for children with elevated BLLs may help reduce lead burden in children. Childhood lead poisoning programs must also move away from relying solely on screening and case management activities and focus on preventing lead exposure through the implementation of housing-based primary prevention.

## Figures and Tables

**Table 1:** Characteristics of the cohort tested for blood lead in Vermont and North Carolina, 1996–1999 (*n* = 996).

Characteristic	No. (%)
Qualifying blood lead level range (μg/dL)	
10–14	452 (45.4)
15–19	315 (31.6)
20–24	112 (11.3)
25–29	59 (5.9)
≥ 30	58 (5.8)
Total	996 (100.0)
Sex	
Male	540 (54.2)
Female	452 (45.4)
Unknown	4 (0.4)
Total	996 (100.0)
Race	
White	397 (39.9)
Black	399 (40.1)
Native American/Alaskan Native	10 (1.0)
Asian/Pacific Islander	10 (1.0)
Hispanic	94 (9.4)
Other	3 (0.3)
Unknown	83 (8.3)
Total	996 (100.0)
State of residence	
Vermont	230 (23.1)
North Carolina	766 (76.9)
Total	996 (100.0)
Sample type at initial blood lead level	
Venous	526 (52.8)
Capillary	432 (43.4)
Unknown	38 (3.8)
Total	996 (100.0)
Location of residence	
Rural	546 (54.8)
Urban	450 (45.2)
Total	996 (100.0)
Age range at qualifying blood lead level (months)	
< 12	52 (5.2)
12–23	491 (49.3)
24–35	199 (20.0)
36–47	110 (11.0)
48–59	81 (8.1)
60–72	63 (6.4)
Total	996 (100.0)

**Table 2:** Unadjusted median time [days (95% CI)] of the time required for BLLs to drop below 10 μg/dL by analysis type and selected covariates, among a cohort of children tested for blood lead in Vermont and North Carolina, 1996–1999.

	Qualifying blood lead level (μg/dL)				
Type of analysis	10–14	15–19	20–24	25–29	≥ 30
Central (*n* = 996)	237 (211–284)	424 (362–479)	659 (593–774)	954 (750–1,057)	1,083 (917–1,083)
Excluding censored children (*n* = 588)	191 (175–216)	267 (224–301)	463 (352–598)	750 (479–954)	723 (151–917)
Interpolated time to end point (*n* = 996)	125 (103–151)	340 (292–401)	564 (483–684)	762 (606–992)	NA
Central, black children (*n* = 399)	265 (211–306)	479 (378–636)	659 (593–863)	957 (750–1,057)	NA
Central, white children (*n* = 397)	230 (194–306)	377 (305–502)	686 (363–823)	1,075 (460–1,075)	1,083 (932–1,083)
Central, male children (*n* = 540)	258 (201–306)	427 (377–566)	598 (559–788)	825 (750–1,057)	NA
Central, female children (*n* = 452)	232 (195–288)	370 (294–476)	686 (509–798)	1,056 (525–1,198)	917 (754–932)
Central, rural address (*n* = 546)	269 (220–316)	440 (355–602)	774 (659–823)	818 (525–957)	1,083 (854–1,083)
Central, urban address (*n* = 450)	214 (182–266)	386 (294–479)	509 (332–598)	1,056 (750–1,198)	NA

NA, not available

**Table 3:** Best predictors of the time required for BLLs to drop below 10 μg/dL overall and by qualifying BLL range, among a cohort of children tested for blood lead from Vermont and North Carolina, 1996–1999.

Variable	β	SE	*p*-Value	HR	95% CI
Overall^a^ (*n* = 996)					
Black	−0.1782	0.0858	0.0378	0.84	0.71–0.99
Male	−0.1859	0.0828	0.0248	0.83	0.71–0.98
Rural address^b^	*—*	*—*	*—*	*—*	*—*
Qualifying BLL: 10–14 μg/dL^c^ (*n* = 452)					
Rural address	−0.1359	0.1101	0.2171	0.87	0.70–1.08
Qualifying BLL: 15–19 μg/dL^c^ (*n* = 315)					
Rural address	−0.2048	0.1575	0.1935	0.82	0.60–1.11
Qualifying BLL: 20–24 μg/dL^c^ (*n* = 112)					
Rural address	−0.7332	0.2883	0.0110	0.48	0.27–0.85
Qualifying BLL: 25–29 μg/dL^c^ (*n* = 59)					
Rural address	0.9427	0.5213	0.0705	2.57	0.92–7.13
Qualifying BLL: ≥ 30 μg/dL^c^ (*n* = 58)					
Rural address	−0.8428	0.6509	0.1954	0.43	0.12–1.54

a Adjusted by qualifying BLL using stratification.

b Association with child’s address differs significantly by qualifying BLL (*p* = 0.04) and is not reported overall.

c Separate analysis within qualifying BLL stratum.
